# Correlated changes in structure and viscosity during gelatinization and gelation of tapioca starch granules

**DOI:** 10.1107/S2052252514019137

**Published:** 2014-10-21

**Authors:** Hsien-Kai Huang, Hwo-Shuenn Sheu, Wei-Tsung Chuang, U-Ser Jeng, An-Chung Su, Wei-Ru Wu, Kuei-Fen Liao, Chun-Yu Chen, Shing-Yun Chang, Hsi-Mei Lai

**Affiliations:** a Department of Chemical Engineering, National Tsing Hua University, Hsinchu 300, Taiwan; b National Synchrotron Radiation Research Center, Hsinchu 300, Taiwan; c Department of Agricultural Chemistry, National Taiwan University, Taipei 106, Taiwan

**Keywords:** tapioca starch granules, gelatinization, gelation, SAXS/WAXS, viscosity

## Abstract

Melting of native tapioca starch granules in aqueous pastes upon heating is observed *in situ* using simultaneous small- and wide-angle X-ray scattering (SAXS/WAXS) and solution viscometry. Correlated structure and viscosity changes suggest closely associated amylose and amylopectin chains in the semicrystalline layers, and the release of amylose chains for enhanced solution viscosity occurs largely after melting of the semicrystalline structure. Before melting, WAXS results reveal mixed crystals of A- and B-types (∼4:1 by weight), whereas SAXS results indicate that the semicrystalline layers are composed of lamellar blocklets of *ca* 43 nm domain size, with polydisperse crystalline (≃7.5 nm) and amorphous (≃1.1 nm) layers alternatively assembled into a lamellar spacing of ≃8.6 nm with 20% polydispersity. Upon melting, the semicrystalline lamellae disintegrate into disperse and molten amylopectin nanoclusters with dissolved and partially untangled amylose chains in the aqueous matrix which leads to increased solution viscosity. During subsequent cooling, gelation starts at around 347 K; successively increased solution viscosity coincides with the development of nanocluster aggregation to a fractal dimension ≃2.3 at 303 K, signifying increasing intercluster association through collapsed amylose chains owing to decreased solvency of the aqueous medium with decreasing temperature.

## Abstract 2

Melting of the semicrystalline structure of native tapioca starch granules is correlated to solution viscosity for elucidating gelatinization and gelation processes.

## 1. Introduction   

Starch for nutritive energy storage in plant roots, tubers or endosperm such as potato, tapioca, corn and rice, comprises primarily carbohydrates in the form of polysaccharides. Previous reviews (Pérez & Bertoft, 2010 ▶; Wang & Copeland, 2013 ▶; Blazek & Gilbert, 2011 ▶; Ratnayake & Jackson, 2009 ▶; Zobel, 1988 ▶) have summarized progress in the understanding of the supramolecular arrangements of starch granules that in nature are adopted for the packing energy harvested from photosynthesis. On the micrometre scale, starch granules ∼1–100 µm in size (Jane *et al.*, 1994 ▶) have a growth-ring structure composed of alternately arranged semicrystalline and amorphous shells (Pérez & Bertoft, 2010 ▶) concentrically developed from the hilum. The semicrystalline shells are mainly composed of clusters of branched-chain amylopectin in contrast to the amorphous shells of long linear-chain amylose and low-molecular-mass amylopectin (Tang *et al.*, 2006 ▶). On the nanometre scale, each semicrystalline shell consists of lamellae of alternating crystalline and amorphous layers, featuring a typical lamellar spacing *L* in the order of 10 nm with crystallinity ranging from 15 to 45%; while the crystalline layer may be further envisioned as oriented aggregates of nanocrystalline ‘blocklets’. At the atomic level, the crystal structure corresponds to either monoclinic (A-type) or hexagonal (B-type) packing of double helices of amylopectin or amylose chains (Zobel, 1988 ▶; Pérez & Bertoft, 2010 ▶). When starch granules are heated in excess water, starch granules swell and disrupt upon melting of the semicrystalline structure in the gelatinization process, resulting in increased solution viscosity. During subsequent cooling, the viscosity further increases upon gelation of the aqueous amylopectin–amylose mixture (Jenkins & Donald, 1998 ▶; Waigh *et al.*, 2000 ▶).

In terms of structural characterization, microbeam X-ray diffraction (Lemke *et al.*, 2004 ▶), time-resolved small-angle and wide-angle X-ray scattering (SAXS/WAXS) (Vermeylen *et al.*, 2006 ▶), small-angle neutron scattering (SANS) (Blazek & Gilbert, 2010 ▶) and three-dimensional structural imaging with second-harmonic generation circular dichroism (Zhuo *et al.*, 2014 ▶) have been used to reveal the nanostructural changes in gelatinization and retrogradation processes of starch granules of various types. Thereby, melting of lamellar and crystalline structures has been revealed in the gelatinization process and fractal-like structural changes in the global semicrystalline structure of starch granules have been revealed from the power-law scattering behavior (Vermeylen *et al.*, 2006 ▶; Lin *et al.*, 2009 ▶). Together with microscopic images, a supramolecular model was proposed for general starch granules (Tang *et al.*, 2006 ▶; Gallant *et al.*, 1997 ▶). However, the location and the state of amylose chains within the granules are not clearly specified in the model; the building blocks (blocklets) of the semicrystalline layers and the corresponding entities upon melting need to be more quantitatively specified for a better understanding of the gelatinization and gelation processes (Pérez & Bertoft, 2010 ▶; Ratnayake & Jackson, 2009 ▶; Tang *et al.*, 2006 ▶).

In this study, the nanocrystalline structural evolution revealed *via* simultaneous SAXS and WAXS for native tapioca starch granules is quantitatively correlated with corresponding changes in solution viscosity. A structural model is then proposed to coherently explain the gelatinization and gelation processes in terms of dissociation and reorganization of amylopectin and amylose chains in structural units of crystalline blocklets and molten nanoclusters.

## 2. Methodology   

### 2.1. Material and measurements   

The characterized native tapioca starch, manufactured by a branch of Ting Hsin Co. in Thailand, contained 12.5% moisture and 21.7% amylose. To ensure an excess water environment throughout the gelatinization and gelation processes, samples with a starch-to-water-weight ratio of 1:20 were used for solution viscometry and those in a weight ratio of 1:3 were used for SAXS and WAXS measurements. Viscosity measurements were performed using an Anton Paar MCR 501 rheometer with a stirring speed of 160 r.p.m. and a temperature program of heating from 313 to 358 K at a rate of 2 K min^−1^, followed by holding at 358 (1) K for 35 min before cooling down (2 K min^−1^) from 358 to 303 K.

Simultaneous SAXS and WAXS measurements were conducted at beamline 23A of the National Synchrotron Radiation Research Center (Jeng *et al.*, 2010 ▶). The sample path length for X-ray scattering was *ca* 1 mm. With an 8.0 keV beam (wavelength λ = 1.55 Å), data were collected simultaneously with a Pilatus 1M-F area detector for SAXS and a flat panel C9728-DK area detector for WAXS during a programmed heating–cooling process similar to that for the viscosity measurements. The sample-to-detector distances were 5102 mm (SAXS) and 119 mm (WAXS) to cover a wide range of the scattering wavevector *q* (= 4πλ^−1^sinθ, where 2θ corresponds to scattering angle). Silver behenate was used for calibrating the *q* values of SAXS data, whereas syndiotactic polystyrene and polyethylene standards were used for WAXS. All data were further corrected for background scattering and transmission loss; reliability was confirmed by repeated runs using fresh specimens.

Tapioca starch granules, after being hydrolyzed with 3.16 *M* H_2_SO_4_ (Angellier *et al.*, 2004 ▶), were imaged with an Inspect S (FEI Co) scanning electronic microscope (SEM) at a working voltage of 15 kV; the semicrystalline layer thicknesses of the starch granules were determined from the SEM images using the *Image J* software (National Institutes of Health, Maryland, USA).

### 2.2. WAXS and SAXS data analysis   

The WAXS data for lamellar crystals in starch granules were analyzed using the Rietveld refinement process (Toby, 2001 ▶; Gualtieri, 2003 ▶). The measured SAXS intensity profiles were analyzed using a scattering model of arrayed disks or platelets for lamellar crystals (Cameron & Donald, 1992 ▶):
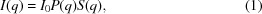
where *I*_0_ is the zero angle (*q* = 0) scattering intensity, *P*(*q*) is the disk form factor and *S*(*q*) is the structure factor; *I*_0_ is contributed by the scattering contrast between the particles and matrix and the number density of the scattering particles (Chen & Lin, 1987 ▶). For homogeneous disk-like particles of radius *R* and thickness *t*_c_, the orientation-averaged form factor is expressed as
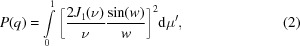
where *J*_1_ is the first-order Bessel function, ν = *qR*(1 − μ′^2^)^1/2^, and *w* = *qt*_c_μ′/2 (Chen & Lin, 1987 ▶). The form factor can be readily modified for polydisperse or elliptic disks (Feigin & Svergun, 1987 ▶). The corresponding structure factor *S*(*q*) for lamellar crystals of alternating crystalline and amorphous layers of long period *L* is described by (Cameron & Donald, 1992 ▶; Richter *et al.*, 1997 ▶)
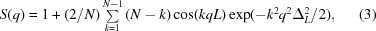
where *N* is the number of stacked lamellae and the fluctuation in long period Δ_*L*_ = [(Δ*t*_c_)^2^ + (Δ*t*_a_)^2^]^1/2^, contributed by polydispersity (fluctuations) in crystalline and amorphous layer thickness (*t*_c_ and *t*_a_, respectively) according to Schultz distribution (Sheu, 1992 ▶).

We note that effects of polydispersity and/or orientational averaging of the form factor would modify the structure factor in equation (1), as detailed in a report by Kotlarchyk & Chen (1983 ▶). Nevertheless, such a correction in *S*(*q*) would be small in our case because of the not large polydispersity in the form factor, and was hence neglected in all the analyses below. Furthermore, the characteristic SAXS lamellar peak from the semicrystalline lamellae of starch granules is determined mainly by X-ray scattering contrast (or electron density difference) between the lamellar crystalline and the amorphous regions, together with the crystal size and number density of the lamellae (*i.e.* crystallinity) and the lamellar packing order (*i.e. N* and Δ_*L*_), as illustrated in equations (1)–(3) [Disp-formula fd3] (also detailed in a previous report by Blazek & Gilbert, 2010 ▶). In principle, the amorphous matrix into which the semicrystalline lamellae embed may carry an electron density different from that of the amorphous zones of the lamellae, leading to a three-phase system. Fortunately, previous studies (Donald *et al.*, 2001 ▶; Blazek & Gilbert, 2010 ▶) indicated that the molecular and electron densities of the water-soaked amorphous regions in semicrystalline lamellae and the matrix of starches are, in general, very close. For simplicity in our SAXS data analyses, we use the same electron density for the matrix and lamellar amorphous zones of tapioca granules before melting.

The SAXS profiles after melting of granules were analyzed using a necklace model (Chen & Teixeira, 1986 ▶; Lin *et al.*, 2009 ▶) for fractal-like aggregates of ellipsoidal primary particles (representing amylopectin nanoclusters), with semi-major axis *A* and semi-minor axis *B*. The orientation-averaged ellipsoidal form factor is
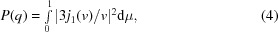
where *v* = *q*[*A* ^2^μ^2^ + *B* ^2^(1 − μ^2^)]^1/2^ and *j*_1_ is the spherical Bessel function of the first-order (Feigin & Svergun, 1987 ▶). The corresponding structure factor
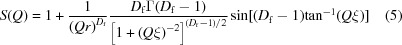
is characterized by the fractal dimension *D*_f_ and the correlation length ξ (Chen & Teixeira, 1986 ▶). The effective radius *r* = (*AB* ^2^)^1/3^ for the primary particles in equation (5) is approximated from the ellipsoid volume on an equivalent-volume basis (Jeng *et al.*, 1999*a* ▶). For a system with coexistence of fractal aggregates and dissociated (disperse) primary particles, scattering contributions from both are assumed linearly additive when using equation (1).

To quantify the level of heterogeneity in a two-phase system, the scattering invariant
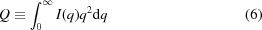
is often used (Feigin & Svergun, 1987 ▶). For convenience when comparing, a relative scattering invariant can be calculated with the upper and lower limits of the integration replaced by the minimum and maximum *q* values of the SAXS *q* range of interest (Su *et al.*, 2008 ▶). Such approximation is appropriate when the *q* range of interest covers major changes in the structural features. Also for convenient comparison, the *relative* crystallinity *X*_c_ is calculated from the WAXS profile based on integrated intensity over *accessible* crystalline reflections and then normalized with respect to the *maximum* value observed, as detailed previously (Su *et al.*, 2009 ▶).

## 3. Result and discussion   

### 3.1. Structural features deduced from SAXS/WAXS analyses   

**Figure 1:**
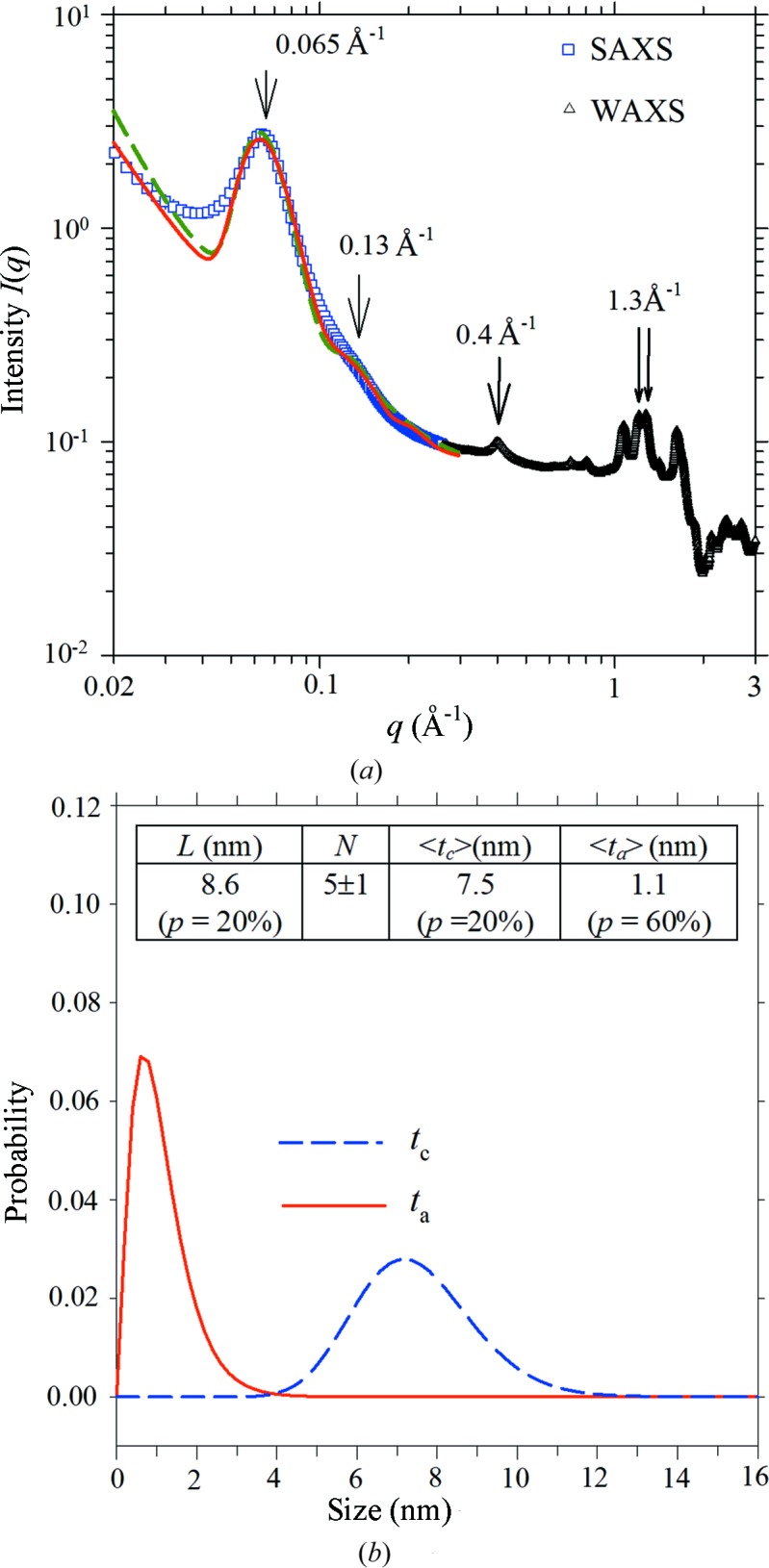
(*a*) Integrated SAXS–WAXS data measured for the native tapioca in excess water. The arrows indicate the lamellar peaks at *q* = 0.065 and 0.13 Å^−1^, the (100)_H_ reflection at *q* = 0.40 Å^−1^ of hexagonal (B-type) crystals, and the characteristic twin peaks near 1.3 Å^−1^ of the monoclinic (A-type) crystals. SAXS data are fitted with the model comprising either arrayed elliptic plates (dotted curve) or disk plates (solid curve). (*b*) Optimal-fit Schultz distribution profiles for thicknesses of the crystalline and amorphous layers in semicrystalline lamellae of the native tapioca. The best-fitted structural parameters with arrayed elliptic plates are shown in the inset, where *p* is the corresponding polydispersity.

Shown in Fig. 1 ▶(*a*) are the combined SAXS and WAXS data for the tapioca starch in excess water at 303 K. The SAXS data reveal a characteristic lamellar peak at *q* = 0.065 Å^−1^, corresponding to a long period *L* ≃ 9.7 nm as estimated from the Bragg law (Imberty & Perez, 1988 ▶), whereas the WAXS data exhibit the characteristic (100)_H_ reflection at *q* = 0.40 Å^−1^ of B-type crystals (Pérez & Bertoft, 2010 ▶) and the twin peaks centered near *q* = 1.3 Å^−1^ of the A-type crystals (Popov *et al.*, 2009 ▶). These results suggest the coexistence of the two crystalline phases in the native tapioca.

Using the model of one-dimensional arrayed elliptic plates illustrated in equations (1)–(3), the SAXS data down to *q* ≃ 0.05 Å^−1^ can be well fitted with the parametric values [*cf*. inset in Fig. 1 ▶(*b*)] of *N* = 5 (1) and *L* = 8.6 nm with 20% polydispersity, hence an average lamellar domain size of *D*_L_ ≃ 43 nm (Vermeylen *et al.*, 2006 ▶); this domain size is also consistent with that estimated from the lamellar peak width using the Scherrer equation. Compared with this refined long period of 8.6 nm with due consideration of the polydispersity, a naïve value of *ca* 10 nm, based only on peak position, is indeed an overestimate (Cameron & Donald, 1992 ▶). Distributions of the crystal thickness with an average *t*_c_ value of 7.5 nm and the amorphous layer with an average *t*_a_ value of 1.1 nm are shown in Fig. 1 ▶(*b*). The fitted values for lateral dimensions of the elliptic plates are *D*_a_ = 26.1 (relatively insensitive parameter) and *D*_b_ = 8.7 nm for the long and the short axes, respectively, with a common 67% polydispersity. We note that the SAXS data fitting may be slightly improved for the second lamellar peak at *ca q* = 0.13 Å^−1^ (Fig. 1*a*) by using arrayed disk plates with a diameter of 7.4 nm (*i.e. D*_a_ = *D*_b_), together with nearly the same values of *N* = 5, *L* = 8.5 nm, *t*_a_ = 1.4 nm and *t*_c_ = 7.0 nm (as compared with that used in the model of arrayed elliptic plates). Nevertheless, the elliptic plates are in better agreement with the WAXS results detailed below. The deviation of the fitting from the data near the second lamellar peak may be improved by using the modified structure factor corrected for polydispersity and anisotropy of the form factor for a smeared oscillation in the calculated *S*(*q*), hence *I*(*q*), profile (Kotlarchyk & Chen, 1983 ▶). Data fitting may be further improved by using different electron densities for the matrix and lamellar amorphous zones ; however, the minor differences should not change much the fitted parameters associated with the structure along the lamellar stacking. Another possibility to improve the data fitting is to use a bimodal distribution to take into account the coexistence of A- and B-type crystals of slightly different sizes revealed from the WAXS data (detailed below). Overall, these parameters can be well mapped into the blocklet model hypothesized previously (Tang *et al.*, 2006 ▶; Pérez & Bertoft, 2010 ▶), in which the semicrystalline layers in starch granules are pictured as self-assembled from oriented blocklets of lamellar crystals.

**Figure 2:**
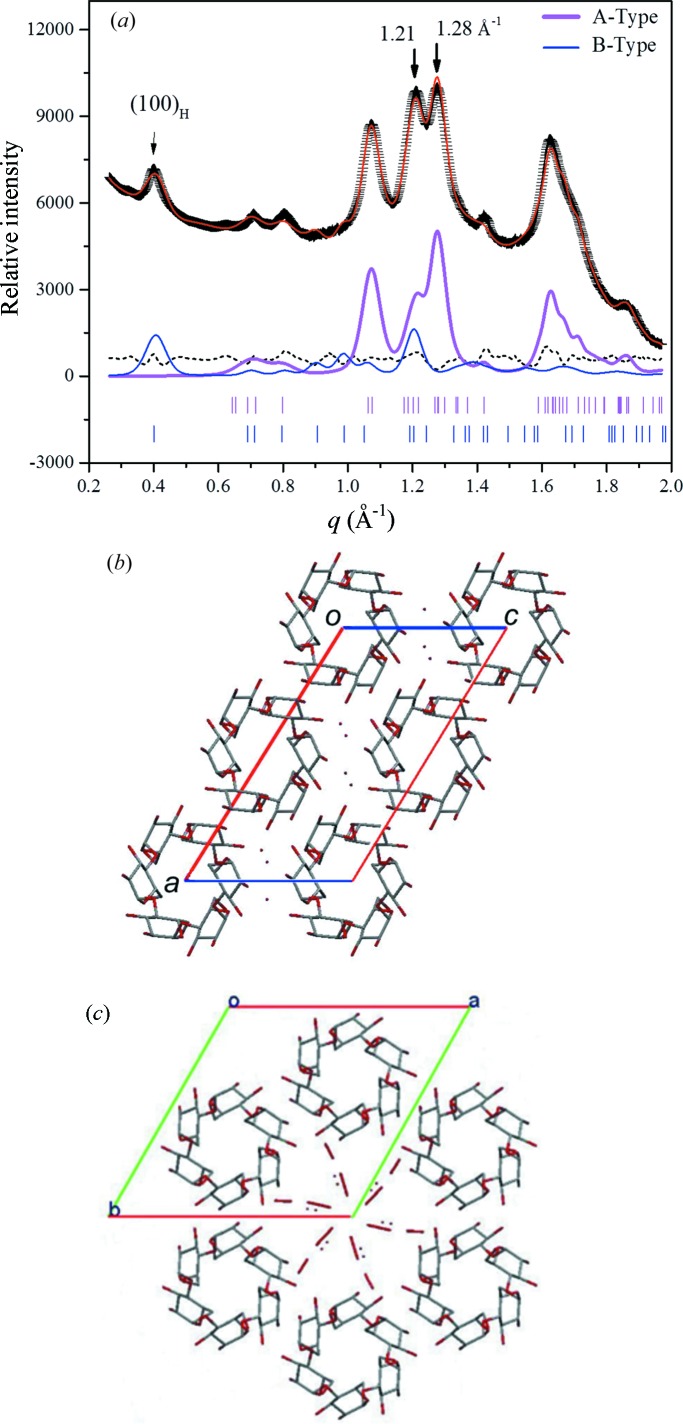
(*a*) Calculated WAXS profile with coexistence of monoclinic and hexagonal crystals in weight ratio of 4:1 gives excellent overlap with experimental data. The upper and lower rows of short rods at the bottom are possible reflections of A-type and B-type crystals, respectively. The dotted curve is the fitting residuals. The arrows mark the (100)_H_ reflection of the hexagonal crystals and the characteristic convoluted peaks at *q* = 1.21 and 1.28 Å^−1^ of the monoclinic crystals. Crystal structure projected along the lamellar stacking direction are shown in (*b*) and (*c*) for the monoclinic and hexagonal phases, respectively. Each type of unit cell contains two pairs of amylopectin or amylose chains in a double-helical conformation; however, the less compactly packed double helices in the latter case leave a water-rich core that accommodates 4.5 times more water (shown as small dots) than does the more densely packed monoclinic phase.

Analysis of WAXS data *via* the Rietveld refinement procedure (Toby, 2001 ▶) with space groups *C*2 for monoclinic (A-type) crystals and *P*6_1_ for hexagonal (B-type) crystals (Popov *et al.*, 2009 ▶; Imberty & Perez, 1988 ▶) led to a satisfactory fit as shown in Fig. 2 ▶(*a*). Corresponding parametric values, summarized in Table 1 ▶, agree well with those reported in the literature. The fitted result suggests coexistence of both monoclinic and hexagonal phases in a weight ratio of 4:1. Illustrated in Figs. 2 ▶(*b*) and 2(*c*) are the fitted structures of monoclinic *C*2 and hexagonal *P*6_1_ crystals projected along the helical axis of amylopectin–amylose chains, *i.e.* the direction of the lamellar stacking (Popov *et al.*, 2009 ▶; Pérez & Bertoft, 2010 ▶). We note that the hexagonally packed helices accommodate a higher water content (Table 1 ▶), and may be relatively permeable to water in an excess-water environment, compared with in the monoclinic packing.

**Figure 3:**
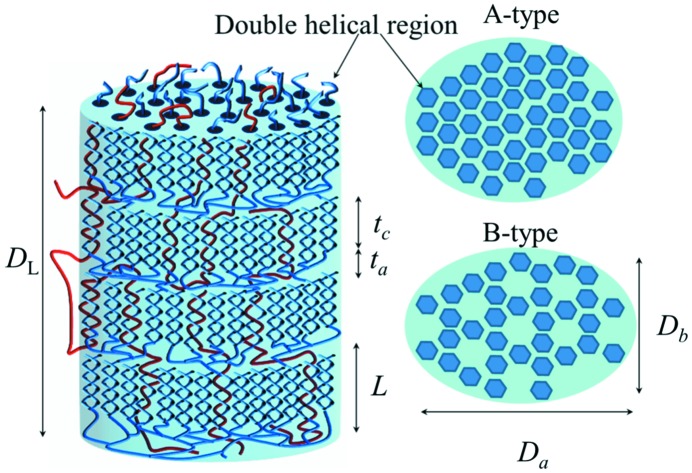
Structural model for the domains (blocklets) of crystalline lamellae in the hierarchical structure of native tapioca granule. The model is characterized by four to five well packed layers of a lamellar domain size (*D*_L_) ≃43 nm, and an idealized elliptic cross section of dimensions *D*_a_ ≃ 26 nm and *D*_b_ ≃ 9 nm (right); the average crystalline and amorphous layer thicknesses (*t*_c_ = 7.5 nm and *t*_a_ = 1.1 nm), accumulate to a lamellar spacing (*L*) of 8.6 nm. More extended and flexible wires (red) are for amylose chains, whereas the branched ones (blue) are for amylopectin chains.

From Fig. 2 ▶(*a*), the characteristic (100)_H_ reflection of the B-type crystals reveals a small crystal dimension of 7.6 nm, as extracted from the peak width based on the Scherrer equation. The global fitting process with the Rietveld refinement, however, reveals a relatively large averaged crystal dimension of *ca* 23 nm (see Table 1 ▶). As large crystals dominate the average size from a global WAXS data fitting, the difference in the global crystal size and specific crystal dimension reveals asymmetric lateral dimensions for the lamellar crystal slabs. This result is more consistent with arrayed elliptic plates (than disks) with a crystal plate thickness *t*_c_ = 7.5 nm along the helical axis from SAXS analysis. Taken together, the observed lamellar crystalline structure may be schematically given, as shown in Fig. 3 ▶, on the basis of the blocklet model proposed previously (Gallant *et al.*, 1997 ▶; Tang *et al.*, 2006 ▶; Pérez & Bertoft, 2010 ▶).

### 3.2. Melting process of the semicrystalline structure   

**Figure 4:**
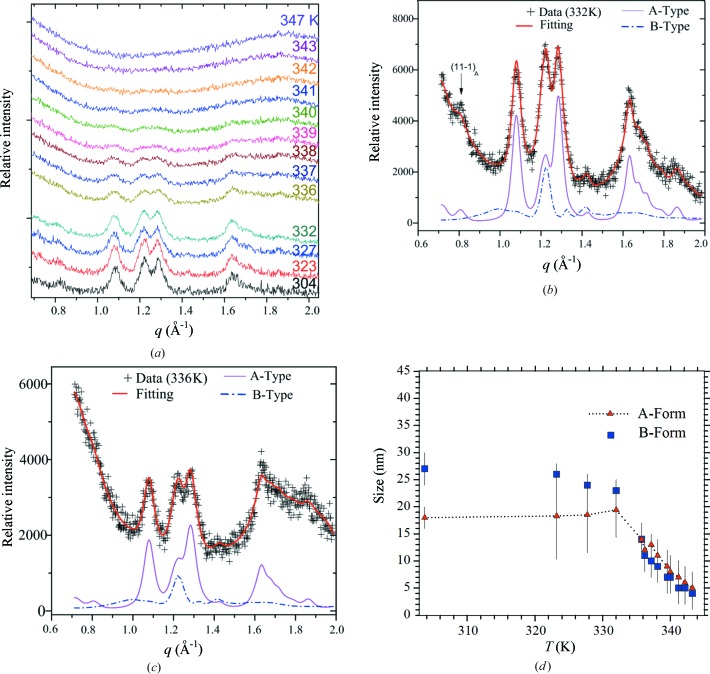
(*a*) WAXS data *in situ* measured for the native tapioca during heating at 2 K min^−1^. Representative data fitting results are shown in (*b*) and (*c*). (*d*) The corresponding evolutions of the averaged A- and B-crystal sizes extracted from data fittings.

The temperature-dependent WAXS data measured during programmed heating of the native tapioca soaked in water are shown in Fig. 4 ▶(*a*). The WAXS peaks start to decay around 328 K and completely disappear after 343 K. Moreover, the intensity ratio of the characteristic twin peaks at *q* = 1.21 and 1.28 Å^−1^ contributed by both A- and B-type crystals (see Fig. 2*a*) remained roughly the same during the melting, implying concomitant melting of the two forms. Following a similar process that was carried out previously, we have fitted all the temperature-dependent WAXS data, as selectively shown in Figs. 4 ▶(*b*) and 4(*c*). The hence obtained evolutions of the crystal sizes are summarized in Fig. 4 ▶(*d*); compared with the B-type crystals, the slower decay of the A-type crystal size suggests slightly better thermal stability, presumably attributable to the lower water content [*cf*. Figs. 2 ▶(*b*) and 2(*c*)]. Previously observed B to A transformation of starch crystallites under pressure also suggested better thermal stability of the A-form (Nishiyama *et al.*, 2010 ▶).

**Figure 5:**
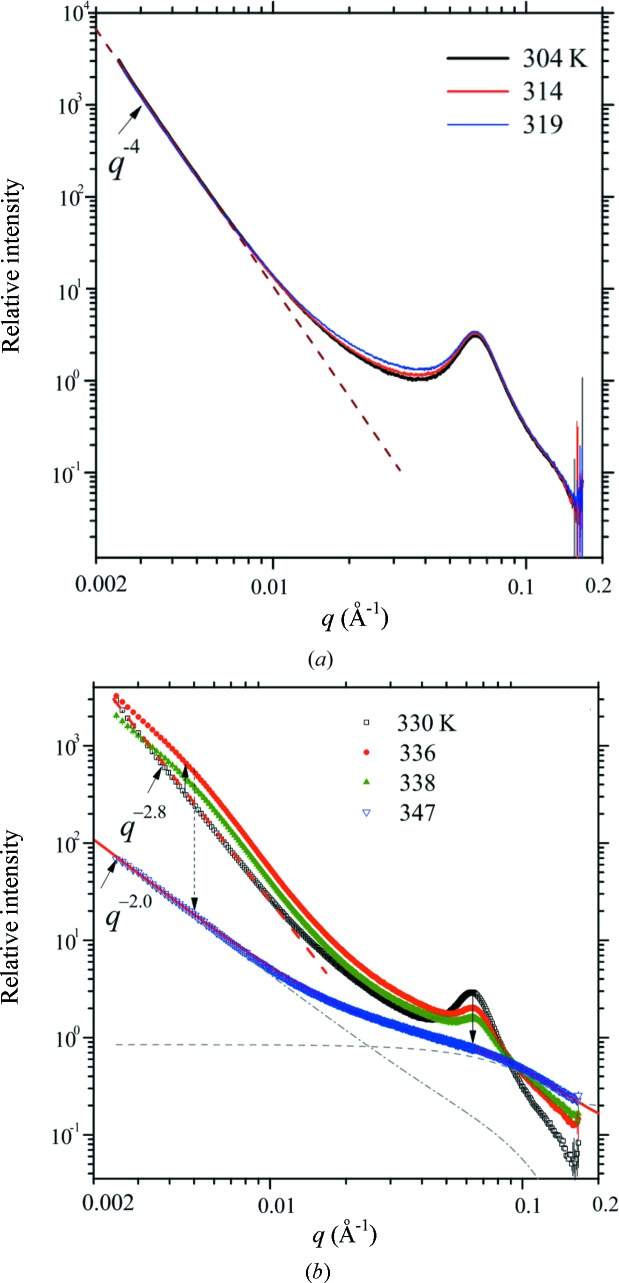
Representative SAXS data measured for the native tapioca during heating: (*a*) below and (*b*) above 323 K. The dotted line in (*a*) marks the common *q* ^−4^ scattering behavior. Vertical arrows in (*b*) emphasize the intensity changes in the low- and high-*q* regions during heating. The arrow outlines the power-law scattering behaviour *I*(*q*) ∝ *q* ^−2.8^ at 330 K; whereas the solid curve calculated using a fractal model describes the fractal scattering behavior *I*(*q*) ∝ *q* ^−2.0^ of the melted structure at 347 K. In (*b*), the long- and short-dashed curves are scattering contributions from the fractal aggregation of nanoclusters and the free nanoclusters, respectively.

The concomitantly obtained SAXS profiles are shown in Fig. 5 ▶, revealing a coherent melting of the lamellar structure with the crystal structure. Below 323 K (Fig. 5 ▶ *a*), there are no significant intensity changes in the higher-*q* region (*q* > 0.05 Å^−1^, dominated by the lamellar structure peak); intensity in the low-*q* region follows a power law of *I*(*q*) ∝ *q* ^−*P*^ with *P* = 4.0, consistent with scattering from smooth surfaces (Schmidt, 1991 ▶; Jeng *et al.*, 1999*b* ▶). The surface scattering feature transits to mass fractal scattering (*P* < 3.0) with *I*(*q*) = *q* ^−2.8^ at 330 K (Fig. 5 ▶ *b*), revealing rupture of ‘blocklets’ of semicrystalline layers. This is followed by the dramatic intensity changes in the low-*q* region (0.003–0.05 Å^−1^) from 336 to 338 K, suggesting disintegration of the semicrystalline layers into discrete ‘debris’ and hence increased heterogeneity in the length-scale at least up to 250 nm (corresponding to the minimum accessible *q* value of 0.0025 Å^−1^); the shoulder around *q* ≃ 0.005 Å^−1^ signifies an intermediate debris dimension or spacing of *ca* 130 nm during the break-down process, as estimated from the Bragg law.

In the high-*q* region of Fig. 5 ▶(*b*), the lamellar peak progressively decays and shifts to slightly higher *q* with increasing temperature before a sharp drop in intensity at 338 K and completely disappearance at 347 K. The discernable shifting of the lamellar peak towards higher-*q* reveals a thinning of the lamellar spacing during melting. In the meanwhile, the decay of the lamellar structure accompanied by an intensity increase in the high-*q* region (>0.1 Å^−1^) suggests melting of lamellar domains into smaller nanoclusters of a noncrystalline entity (as revealed from the corresponding WAXS profiles). These smaller structural features (*i.e.* nanoclusters or debris of disintegrated lamellae) dominate the scattering contribution in the corresponding higher-*q* regime (Feigin & Svergun, 1987 ▶).

**Figure 6:**
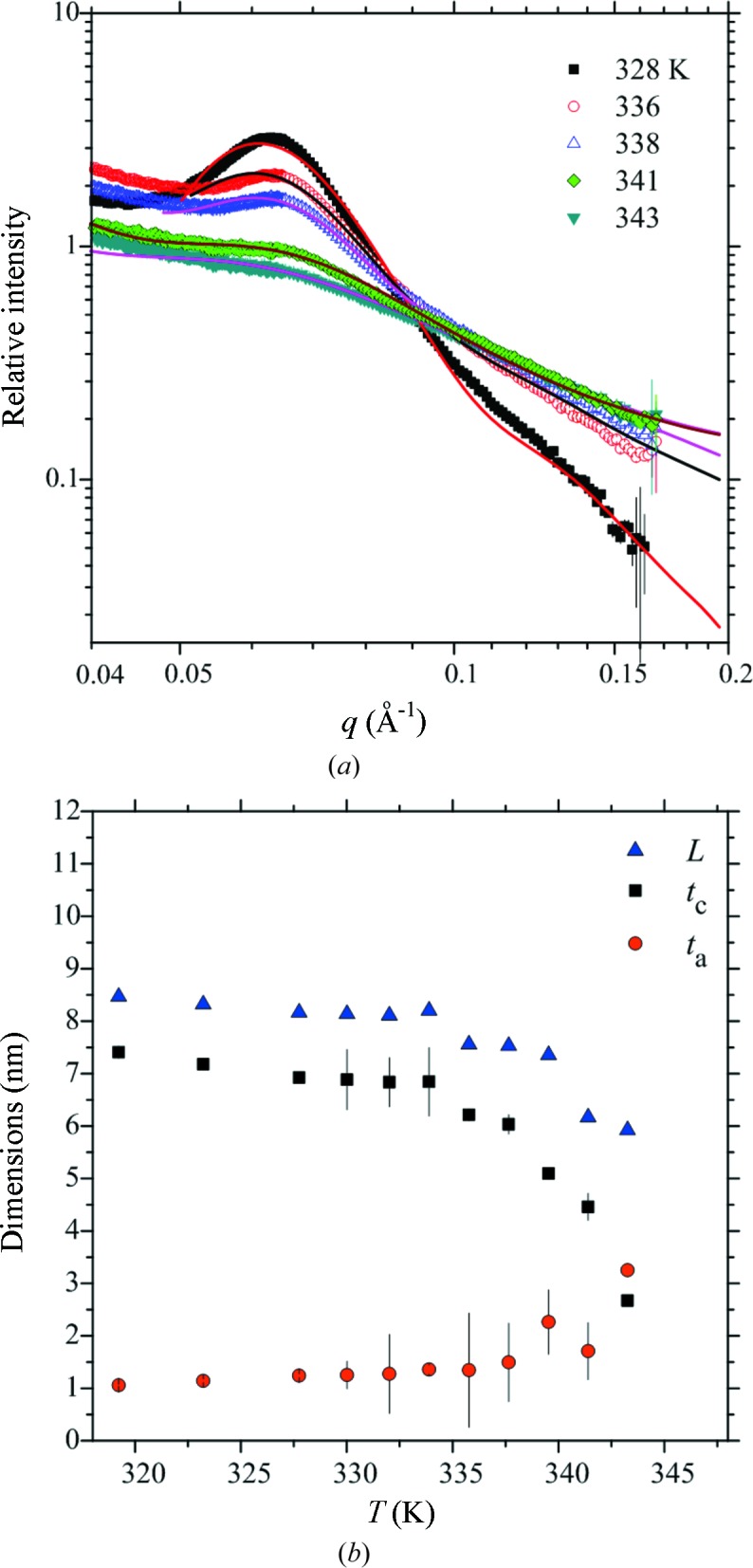
(*a*) Representing SAXS data fitting (solid curves) for the *in situ* SAXS data of the native tapioca starch during the heating process. (*b*) Corresponding parameters used in the fitting, including the lamellar spacing (*L*) and the crystal and amorphous layer thicknesses (*t*_c_ and *t*_a_).

**Figure 7:**
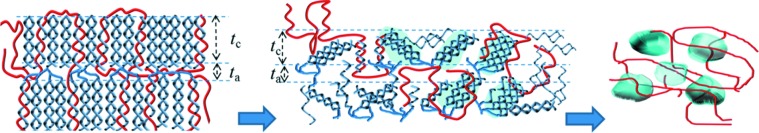
The melting process of the semicrystalline lamellae. Schematically shown (left) are stacked lamellae of well packed crystalline nanoclusters of amylopectin (blue) and amylose (red) chains. Melting of defect-rich regions leads to partial loss of integrity of the lamellae, into layers alternatingly rich and poor in tilted and partially disordered nanoclusters (shaded background in blue), as shown in the middle. The process ends with disperse and molten nanoclusters (bright blue) of amylopectin loosely associated with relatively untangled linear amylose chains (right).

Using the same model of arrayed platelets adopted for construction of Fig. 1 ▶, we can fit all *in situ* SAXS data (from 303 to 343 K) in the *q* range of 0.05–0.14 Å^−1^, where the scattering intensity is dominantly contributed by the lamellar structure (Fig. 6 ▶ *a*). The parameter hence extracted including the lamellar spacing *L* along with the crystal and amorphous layer thickness (*t*_c_ and *t*_a_) are summarized in Fig. 6 ▶(*b*). These temperature-dependent structural parameters serve as indicators to the disintegration process of the lamellar structure. According to Fig. 6 ▶(*b*), *t*_c_ starts to decrease continuously from 7.2 nm at 323 K to 2.7 nm at 343 K, before vanishing together with the crystal melting (Fig. 4 ▶). However, the corresponding increase in *t*_a_ (2.1 nm) from 1.1 to 3.2 nm is only about half of the decrease in *t*_c_ (4.5 nm), suggesting that not all chains of melted crystals resided in the amorphous layer. Instead, a substantial number of the melted chains, most likely untangled amylose, were released from the lamellar structure. A similar suggestion was also proposed (Saibene & Seetharaman, 2010 ▶; Bahnassey & Breene, 1994 ▶) on the basis of drastically enhanced solution viscosity observed close to the end of gelatinization. The consequently evacuated space may lead to tilting of the residual crystalline chains for a reduced *t*_c_ value, with a relatively stable *t*_a_ value, as illustrated in Fig. 7 ▶.

At 347 K, with all the crystalline and lamellar structures being completely melted (*cf*. Figs. 4 ▶ and 5 ▶), the SAXS data measured can be satisfactory fitted (Fig. 5 ▶ *b*) with fractal aggregates of ellipsoidal nanoclusters coexisting with free nanoclusters, as described by equations (4) and (5). The fitted parameters include a fractal dimension *D*_f_ = 2.0 (0.05) and ellipsoid nanoclusters with major axis 2*A* = 7.1 (0.7) nm and minor axis 2*B* = 2.6 (0.3) nm; whereas the fractal structure size is only insensitively determined to be larger than 250 nm, due to the accessible *q* range (of only a power-law scattering regime without a transition zone to form-factor scattering). Amylopectin nanoclusters of similar size and shape were also proposed previously as a basic construction unit for the semicrystalline layers (Pérez & Bertoft, 2010 ▶; Bertoft, 2004 ▶). Moreover, the fitted population of the free nanoclusters is an order-of-magnitude higher than that in the fractal-like aggregates, as revealed from the respective high-*q* contributions in Fig. 5 ▶(*b*). The disjointed amylopectin nanoclusters and released amylose chains would undergo gelation in the subsequent cooling process, as detailed below.

### 3.3. Correlated viscosity and structural changes in gelatinization   

**Figure 8:**
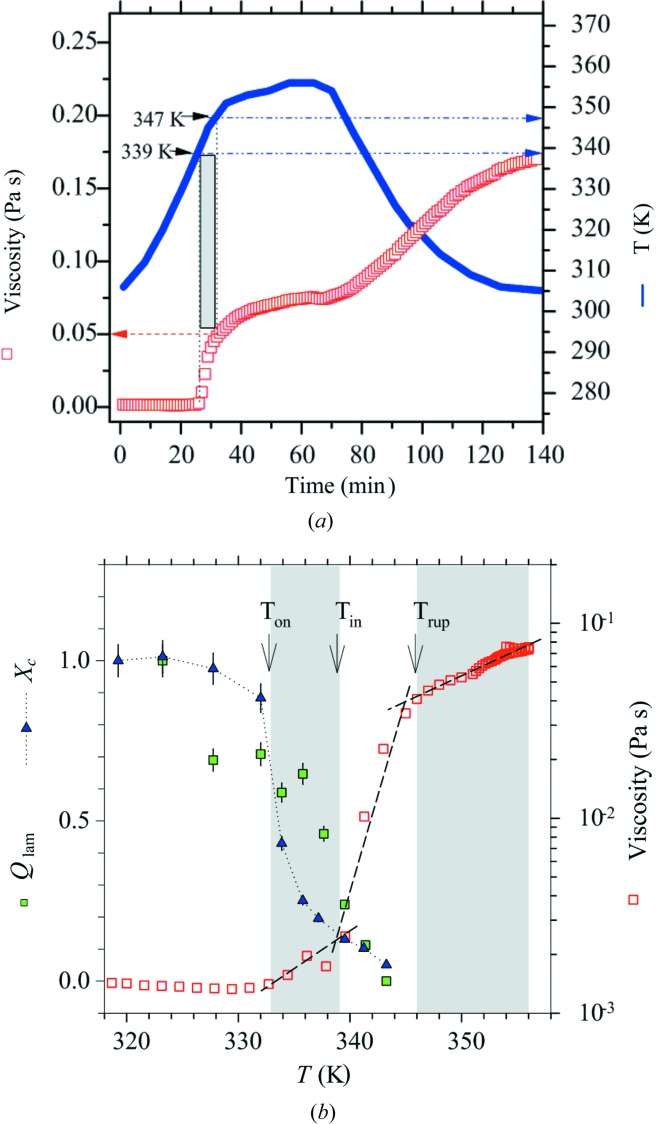
(*a*) Viscosity profile measured for the native tapioca starch in the thermal treatment indicated. The grey zone marks the onset increase of viscosity between 339 and 347 K. (*b*) Correlated structure and viscosity changes in the heating (gelatinization) process, marked with *T*_on_, *T*_in_ and *T*_rup_ for characteristic transitions.

We have also measured drastic changes in the solution viscosity (see Fig. 8 ▶ *a*) of the native tapioca in a similar heating process as that used for the SAXS and WAXS measurements. The viscosity was initially very low but sharply increased to *ca* 0.05 Pa s in the melting range of 339–347 K, indicating gelatinization of the native tapioca starch granules (Bahnassey & Breene, 1994 ▶). With further heating to and holding at ∼358 K, the viscosity increased only modestly and leveled at ∼0.07 Pa s. During the subsequent cooling from 358 to 303 K for gelation, viscosity further increased strongly to 0.17 Pa s (Brouillet-Fourmann *et al.*, 2003 ▶).

The correlated scattering invariant *Q*_lam_, the relative crystallinity *X*_c_, and the viscosity profiles observed with a similar heating process for the native tapioca granules are shown in Fig. 8 ▶(*b*). Above 328 K, *Q*_lam_ calculated from the scattering *q* range dominated by the lamellar structure (0.05–0.16 Å^−1^ in Fig. 6 ▶ *a*) is found to decrease with *X*_c_, but only after substantial decays of these two quantities at an onset temperature *T*_on_ ≃ 333 K the viscosity starts to increase modestly. At an induction temperature *T*_in_ = 339 K, with both *Q*_lam_ and *X*_c_ largely reduced to one-tenth of the initial values, an upturn of viscosity is observed. After the blocklet rupture temperature *T*_rup_ ≃ 345 K where both *Q*_lam_ and *X*_c_ have largely vanished, the viscosity increases at a significantly lower rate. The close correspondence among changes in *Q*_lam_, *X*_c_ and viscosity strongly suggest a close association of amylopectin and amylose in the semicrystalline lamellae, and a release of the amylose chains for an increase of viscosity in gelatinization occurs largely after melting of the lamellar structure.

### 3.4. Gelation with nanoclusters   

**Figure 9:**
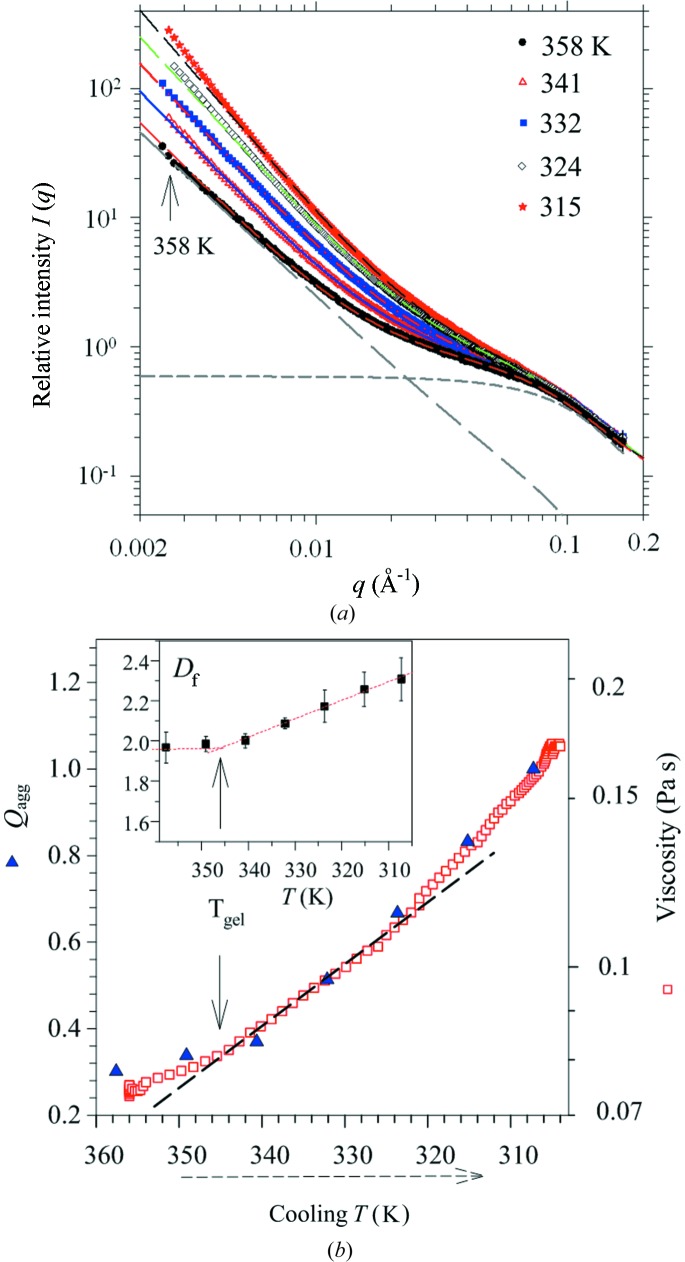
(*a*) SAXS profiles measured during the cooling process. Data are fitted (dashed curves) using a model of coexisting fractal aggregates with their primary particles; the respective contributions are selectively illustrated with the long- and short-dashed curves below for the 358 K case. (*b*) Correlated changes of viscosity and the SAXS invariant *Q*_agg_ contributed from the fractal aggregates with increasing fractal dimension *D*_f_ (inset). *T*_gel_ marks the temperature for coherently enhanced transitions in structure and viscosity.

Compared with that in the heating process for gelatinization, the evolution of WAXS profiles observed during the subsequent cooling for gelation are fairly simple and monotonous. These WAXS profiles, resembling to that at 347 K in Fig. 4 ▶(*a*), are largely featureless, revealing no obvious crystalline structure. In contrast, the concomitantly measured SAXS profiles (Fig. 9 ▶ *a*) exhibit drastic and successive changes in the low-*q* regime (0.0025–0.02 Å^−1^), revealing development of *meso*-structure in gelation during cooling. Using the same model of coexisting fractal aggregates of the nanoclusters and individual nanoclusters as that used previously (*cf*. Fig. 5 ▶ *b*), we could satisfactorily fit all the SAXS profiles with ellipsoidal nanoclusters with constant major axis 2*A* = 7.2 nm and minor axis 2*B* = 2.6 nm. The fitted parameters reveal a steady growth of the fractal aggregates, in terms of increases in the fractal dimension and population of the aggregate, at the expense of the free nanoclusters of a correspondingly decayed population. As illustrated in the inset of Fig. 9 ▶(*b*), fractal dimension *D*_f_ of the fractal aggregates increases progressively from 2.0 at 358 K to 2.3 at 315 K, indicating increasingly condensed fractal aggregates (Schmidt, 1991 ▶). The fractal size extracted from the fitted correlation ξ values amounts to a micrometre scale (Chen & Teixeira, 1986 ▶); owing to the limited low-*q* data manifesting only power scattering behavior without a cut-off, the fractal aggregation sizes are only insensitively determined, and serve mainly as a lower bound at best (Jeng *et al.*, 1999*b* ▶). The growth behavior of the fractal aggregates, however, can be represented by the corresponding scattering invariant *Q*_agg_, separated from the SAXS data fitting as illustrated in Fig. 9 ▶. As a result, highly coherent changes in the structural parameter *Q*_agg_, fractal dimension *D*_f_ and the solution viscosity, as elucidated in Fig. 9 ▶(*b*), reveal a strong correlation of the nanocluster aggregation to the viscosity change, with a consistent break in each of the three curves signifying a characteristic temperature *T*_gel_ for gelation.

**Figure 10:**
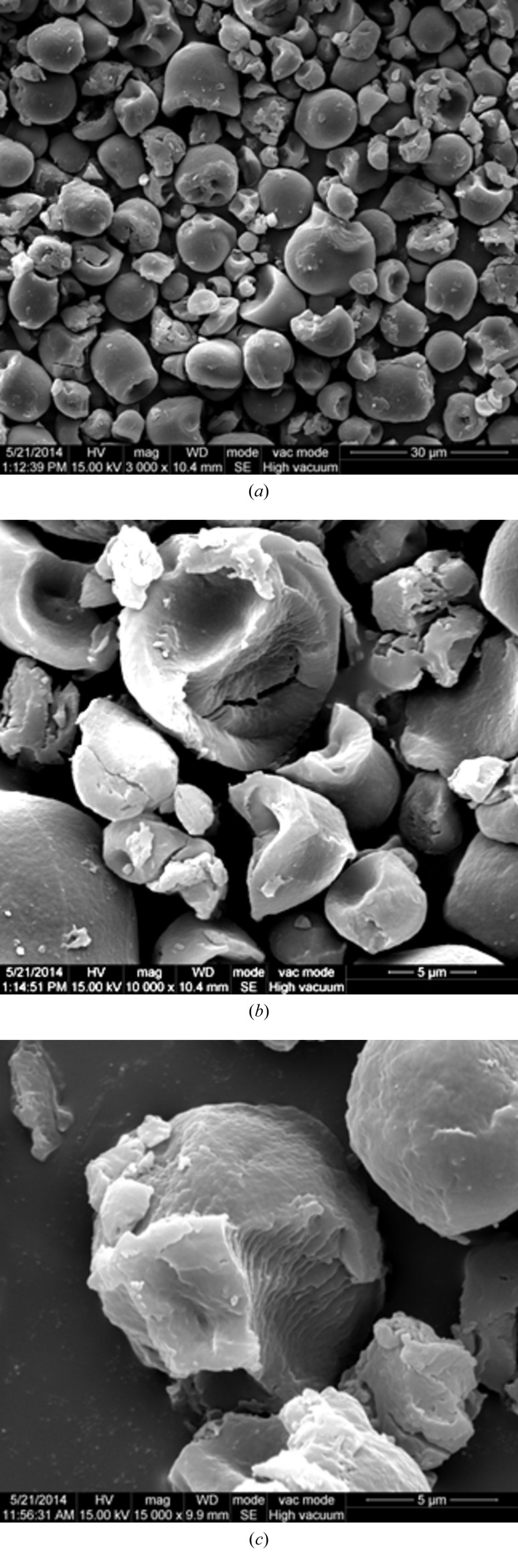
SEM images for tapioca starch granules (2–35 µm) with the characteristic truncated globular shape after hydrolysis with acid for two (*a*, *b*) and three (*c*) days, revealing successively decayed shell-layer thicknesses (*cf*. the truncated granules near the centre) of 92 (14) nm and 69 (9) nm, respectively.

**Figure 11:**
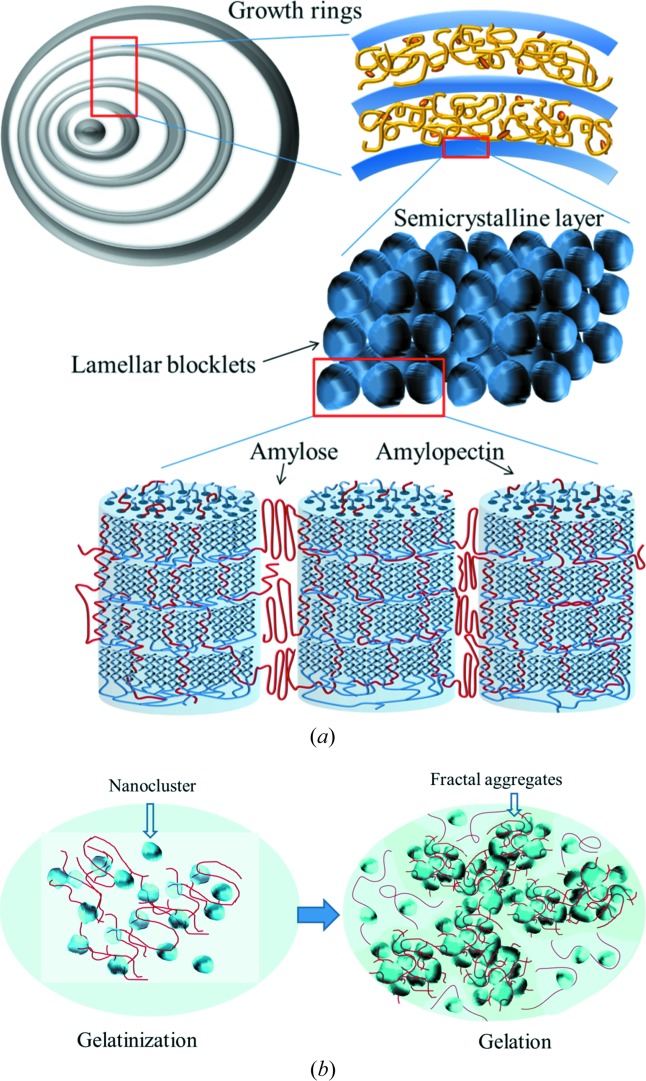
Cartoons for a hierarchical structure model of the native tapioca starch granule (*a*). (*b*) Gelatinization for disperse linear amylose chains and ellipsoidal nanoclusters of amylopectin, of dimensions *ca* 7 × 3 × 3 nm (left); gelation for loosely fractal-structured networks on a micrometer scale (right).

SEM images for the tapioca starch granules with characteristic truncated globular shape and the fractural sections of hydrolyzed starch granules are shown in Fig. 10 ▶. From which, average semicrystalline layer thicknesses of 92 (14) nm and 69 (9) nm are extracted for the samples subjected to two and three days of hydrolysis, respectively. Based on the hydrolysis rate of the tapioca starch observed over ten days, an original semicrystalline layer thickness is extrapolated to be around 140–200 nm, corresponding roughly to three to five stacked blocklets (considering a lamellar domain size *ca* 43 nm as extracted previously). With due considerations of all the observed structural features–roles of blocklets, amylopectin nanoclusters and amylose chains, we propose in Fig. 11 ▶ a comprehensive picture of the observed gelation process in cooling and the previous gelatinization process in heating of the native tapioca starch granules. In this model, the semicrystalline layers comprise blocklets interwoven with long-chain linear amylose. The outer and larger semicrystalline layers in the starch granule would contain more blocklets that require more amylose chains for space filling. Such a consequence is consistent with the general feature that more amylose chains are found in the outer zone (of larger growth rings) or near the surface of starch granules (Pérez & Bertoft, 2010 ▶). With the proposed picture, the gelatinization and gelation processes may be coherently explained as: dissociation of blocklets in the semicrystalline layers into noncrystalline nanoclusters followed by releasing amylose chains into the aqueous matrix during heating, and reorganization of the nanoclusters of amylopectin and amylose chains into fractal-like aggregates upon cooling.

## 4. Conclusions   

Our SAXS/WAXS observations have elucidated concomitant occurrence of melting of lamellar nanocrystals, disintegration of the constituting blocklets, and the resulting dissipation of the semicrystalline layers upon gelatinization during heating in excess water to 333–347 K. The solution viscosity increases rapidly only after complete dissipation of the semicrystalline structure, suggesting that amylose chains are initially trapped by cocrystallization with amylopectin in blocklets of 40–50 nm. The gelatinization process corresponds to melting of nanocrystals and disintegration of blocklets into prolate nanoclusters of molten amylopectin, releasing amylose chains into the aqueous matrix. The gelation process upon subsequent cooling may then be coherently attributed to aggregation of noncrystalline amylopectin nanoclusters connected by amylose chains into fractal-structured networks of gradually increasing fractal dimension (*D*_f_) from 2.0 at 347 K to 2.3 at 303 K.

## Figures and Tables

**Table 1:** Rietveld refinement parameters for the WAXS data of the native tapioca in excess water *N*_w_ is the number of water molecules in a unit cell of volume *V*_i_, *W*_i_ is the weight percentage of A- or B-type crystals and *H* the average crystal size. The *N*_w_ values were taken from the previous crystal structures and kept fixed during structural refinement.

	Space group	Cell parameters (, )	*V*_i_ (^3^)	*N*_w_	*W*_i_ (%)	*H* (nm)
A-type	*C*2	*a* = 21.171(8), *b* = 10.788(9), *c* = 11.776(3), = 122.81(3)	2260(2)	8	79.0(9)	18.3
B-type	*P*6_1_	*a* = *b* = 18.23(2), *c* = 11.06(3)	3184(7)	36	21.0(3)	22.7

## References

[bb1] Angellier, H., Choisnard, L., Molina-Boisseau, S., Ozil, P. & Dufresne, A. (2004). *Biomacromolecules*, **5**, 1545–1551. 10.1021/bm049914u 15244476

[bb2] Bahnassey, Y. A. & Breene, W. M. (1994). *Starch*, **46**, 134–141.

[bb3] Bertoft, E. (2004). *Carbohydr. Polym.* **57**, 211–224.

[bb5] Blazek, J. & Gilbert, E. P. (2010). *Biomacromolecules*, **11**, 3275–3289. 10.1021/bm101124t 21033657

[bb4] Blazek, J. & Gilbert, E. P. (2011). *Carbohydr. Polym.* **85**, 281–293.

[bb6] Brouillet-Fourmann, S., Carrot, C. & Mignard, N. (2003). *Rheol. Acta*, **42**, 110–117.

[bb7] Cameron, R. E. & Donald, A. M. (1992). *Polymer*, **33**, 2628–2635.

[bb8] Chen, S.-H. & Lin, T.-L. (1987). *Methods of Experimental Physics: Neutron Scattering in Condensed Matter Research*, Vol. 23B, edited by K. Sköld & D. L. Price, ch. 16. New York: Academic Press.

[bb9] Chen, S. H. & Teixeira, J. (1986). *Phys. Rev. Lett.* **57**, 2583–2586. 10.1103/PhysRevLett.57.2583 10033804

[bb10] Donald, A. M., Kato, K. L., Perry, P. A. & Waigh, T. A. (2001). *Starch*, **53**, 504–512.

[bb11] Feigin, L. A. & Svergun, D. I. (1987). Structure Analysis by Small-Angle X-ray and Neutron Scattering, p. 69. New York: Plenum.

[bb12] Gallant, D. J., Bouchet, B. & Baldwin, P. M. (1997). *Carbohydr. Polym.* **32**, 177–191.

[bb13] Gualtieri, A. F. (2003). A guided training exercise of quantitative phase analysis using EXPGUI. *GSAS* Tutorials and Examples. http://www.CCP14.ac.uk/solution/gsas/index.html.

[bb14] Imberty, A. & Perez, S. (1988). *Biopolymers*, **27**, 1205–1221.

[bb15] Jane, J.-L., Kasemsuwan, T., Leas, S., Zobel, H. & Robyt, J. F. (1994). *Starch*, **46**, 121–129.

[bb16] Jeng, U., Lin, T.-L., Tsao, C.-S., Lee, C.-H., Wang, L. Y., Chiang, L. Y. & Han, C. C. (1999*a*). *J. Phys. Chem. B*, **103**, 1059–1066.

[bb17] Jeng, U., Liu, W.-J., Lin, T.-L., Wang, L. Y. & Chiang, L.-Y. (1999*b*). *Fullerene Sci. Technol.* **7**, 599–608.

[bb18] Jeng, U.-S. *et al.* (2010). *J. Appl. Cryst.* **43**, 110–121.

[bb19] Jenkins, P. J. & Donald, A. M. (1998). *Carbohydr. Res.* **308**, 133–147.

[bb20] Kotlarchyk, M. & Chen, S. H. (1983). *J. Chem. Phys.* **79**, 2461–2469.

[bb21] Lemke, H., Burghammer, M., Flot, D., Rössle, M. & Riekel, C. (2004). *Biomacromolecules*, **5**, 1316–1324. 10.1021/bm0499536 15244446

[bb22] Lin, J.-M., Lin, T.-L., Jeng, U., Huang, Z.-H. & Huang, Y.-S. (2009). *Soft Matter*, **5**, 3913–3919.

[bb23] Nishiyama, Y., Putaux, J. L., Montesanti, N., Hazemann, J.-L. & Rochas, C. (2010). *Biomacromolecules*, **11**, 76–87. 10.1021/bm900920t 19994877

[bb24] Pérez, S. V. & Bertoft, E. (2010). *Starch*, **62**, 389–420.

[bb25] Popov, D., Buléon, A., Burghammer, M., Chanzy, H., Montesanti, N., Putaux, J., Potocki-Véronèse, G. & Riekel, C. (2009). *Macromolecules*, **42**, 1167–1174.

[bb26] Ratnayake, W. S. & Jackson, D. S. (2009). *Adv. Food Nutr. Res.* **55**, 222–260. 10.1016/S1043-4526(08)00405-1 18772106

[bb27] Richter, D., Schneiders, D., Monkenbusch, M., Willner, L., Fetters, L. J., Huang, J. S., Lin, M., Mortensen, K. & Farago, B. (1997). *Macromolecules*, **30**, 1053–1068.

[bb28] Saibene, D. & Seetharaman, K. (2010). *Carbohydr. Polym.* **82**, 376–383.

[bb29] Schmidt, P. W. (1991). *J. Appl. Cryst.* **24**, 414–435.

[bb30] Sheu, E. Y. (1992). *Phys. Rev. A*, **45**, 2428–2438. 10.1103/physreva.45.2428 9907265

[bb31] Su, C. H., Jeng, U., Chen, S. H., Lin, S. J., Ou, Y. T., Chuang, W. -T. & Su, A. C. (2008). *Macromolecules*, **41**, 7630–7636.

[bb32] Su, C. H., Jeng, U., Chen, S. H., Lin, S. J., Wu, W. R., Chuang, W. T., Tsai, J. C. & Su, A. C. (2009). *Macromolecules*, **42**, 6656–6664.

[bb33] Tang, H., Mitsunaga, T. & Kawamura, Y. (2006). *Carbohydr. Polym.* **63**, 555–560.

[bb34] Toby, B. H. (2001). *J. Appl. Cryst.* **34**, 210–213.

[bb35] Vermeylen, R., Derycke, V., Delcour, J. A., Goderis, B., Reynaers, H. & Koch, M. H. J. (2006). *Biomacromolecules*, **7**, 1231–1238. 10.1021/bm050651t 16602743

[bb36] Waigh, T. A., Gidley, M. J., Komanshek, B. U. & Donald, A. M. (2000). *Carbohydr. Res.* **328**, 165–176. 10.1016/s0008-6215(00)00098-7 11028784

[bb37] Wang, S. & Copeland, L. (2013). *Food Funct.* **4**, 1564–1580. 10.1039/c3fo60258c 24096569

[bb38] Zhuo, G.-Y., Lee, H., Hsu, K. J., Huttunen, M. J., Kauranen, M. Y., Lin, Y.-Y. & Chu, S. W. (2014). *J. Microsc.* **253**, 183–190. 10.1111/jmi.12108 24392849

[bb39] Zobel, H. F. (1988). *Starch*, **40**, 1–7.

